# Modulation of miR-429 during osmotic stress in the silverside *Odontesthes humensis*

**DOI:** 10.3389/fgene.2022.903201

**Published:** 2022-09-07

**Authors:** Antônio D. Pagano, Bruna F. Barreto, William B. Domingues, Tony L. R. Silveira, Leandro S. Nunes, Eduardo B. Blodorn, Eduardo N. Dellagostin, Mariana H. Remião, Ricardo B. Robaldo, Vinicius F. Campos

**Affiliations:** ^1^ Laboratório de Genômica Estrutural, Programa de Pós-Graduação em Biotecnologia, Centro de Desenvolvimento Tecnológico, Universidade Federal de Pelotas, Pelotas, Rio Grande do Sul, Brazil; ^2^ Instituto de Ciências Biológicas, Universidade Federal do Rio Grande, Rio Grande, Rio Grande do Sul, Brazil; ^3^ Instituto de Biologia, Universidade Federal de Pelotas, Pelotas, Rio Grande do Sul, Brazil

**Keywords:** microRNA, osmoregulation, pejerrey, aquaculture, salinity

## Abstract

Silverside fish inhabit marine coastal waters, coastal lagoons, and estuarine regions in southern South America. Although silversides are not fully adapted to freshwater, they can tolerate a wide range of salinity variations. MicroRNAs (miRNAs) are a class of ∼22 nucleotide noncoding RNAs, which are crucial regulators of gene expression at post-transcriptional level. Current data indicate that miRNAs biogenesis is altered by situations of environmental stress, thereby altering the expression of target mRNAs. Foremost, the silversides were acutely exposed to 30 g.L^−1^ of salt to reveal in which tissue miR-429 could be differentially expressed. Thus, fish were acclimated to freshwater (0 g.L^−1^) and to brackish water (10 g.L^−1^), and then exposed to opposite salinity treatment. Here, we reveal that miR-429, a gill-enriched miRNA, emerges as a prime osmoregulator in silversides. Taken together, our findings suggest that miR-429 is an endogenous regulator of osmotic stress, which may be developed as a biomarker to assist silverside aquaculture.

## Introduction

Salinity is one of the main abiotic properties that determine the distribution of fishes across aquatic environments. When faced with salinity variations, to maintain the osmotic balance, fishes depend on osmotically-induced responses, and systemic endocrine signaling induced by ionic transport in the gills, to salt-stress adaptation (Seale and Breves, 2022). In this sense, *Odontesthes* spp., which are popularly known as pejerreyes or silversides, form the most diverse genus in the Atherinopsidae group, with species inhabiting marine coastal waters, coastal lagoons, and estuarine regions in southern South America (Dyer, 2006). Currently, even though all *Odontesthes* spp. have a common marine origin, some fishes from these species currently occupy freshwater (FW) environments (Campanella et al., 2015). Transitions between FW and marine habitats are related with increased species richness. Indeed, FW adaptations are shaped by evolutionary patterns, and are essentially involved with osmoregulatory adjustments because FW and diadromous lineages tolerate high salinity (Mank and Avise, 2006).

The silverside *Odontesthes humensis* is an endemic species that inhabits coastal lagoons, especially from southern Brazil, Uruguay, and Argentina (Bemvenuti, 2006). More specifically, fish species that inhabit environments where there are continuous alterations in water salinity are regarded as euryhaline, and are therefore able to adapt to osmotic stress by several efficient mechanisms of osmosensing and osmotic stress signaling (Fiol and Kültz, 2007). Given that silversides occupy both marine and FW environments, they present this interesting ability to tolerate salt. Naturally, they constantly face variations in salinity in the environment. Thus, these fish have developed excellent mechanisms of osmotic adaptation and appear to be excellent bioindicators in environmental studies due to their demand for good water quality, surviving only in a narrow range of water parameters (Zebral et al., 2017; Silveira et al., 2018a; Martins et al., 2021).

In this regard, the FW silversides euryhalinity has a considerable application for the aquaculture of these species in estuarine regions and in brackish environments that are characterized by significant salinity variations (Piedras, 2009). Salinity is a frequent abiotic stressor that restricts fish growth and development, and favors the impairment of the existing macromolecules, such as proteins, mRNAs, DNA, and lipids (Bartel, 2009). Thus, when faced with salinity variations, fishes employ many physiological acclimations to rapidly respond to osmotic stress, such as the induction of molecular chaperones, rapid clearance of damaged macromolecules, growth arrest, and the alteration of gene expression of multiple genes that mediates osmotic stress tolerance (Fiol and Kültz, 2007; Richter and Haslbeck, 2010; Silveira et al., 2018b). This gene expression modulation to maintain the osmotic balance may be directly influenced by miRNAs (Yan et al., 2012a).

In this sense, miRNAs are a class of noncoding RNA molecules, 22–25 nucleotides in length, which negatively regulate gene expression at the post-transcriptional level (Makeyev and Maniatis, 2008). In the cytoplasm, after two-step cleavage of primary miRNA and precursor miRNA, mature miRNA is loaded by Argonaute 2 protein (AGO2), which compounds the miRNA-Induced Silencing Complex (miRISC). The miRNA guides this effector complex to target sites in the 3’ untranslated region (UTR) of mRNAs. The silencing mechanism is based on the complementarity levels between miRNA in miRISC and mRNA, and can be cleavage of target mRNA or inhibition of its translation (Gebert and Macrae, 2019).

Based on miRNAs’ regulatory function and rapid modulation during stresses, they could act as biomarkers for biotic and abiotic stress because adverse environmental situations, (e.g., osmotic stress) can shape the biogenesis of miRNAs, the expression of target mRNAs, and the miRNA-protein complexes activity (Leung and Sharp, 2010). More specifically, miR-429 is considered to be a biomarker of salinity because it participates in a complex regulatory circuit that is responsible for both osmolality and ionic balance of plasma, as previously shown in tilapia during exposition to salinity of 20 g.L^−1^ (Yan et al., 2012b). Meanwhile, miR-429 has already been established to be a crucial osmotic regulator in euryhaline species. Therefore, we propose to analyze its relative expression during situations of environmental salinity.

This study aims to prospect miR-429 as an epigenetic biomarker of environmental salinity in the silverside *Odontesthes humensis* under osmotic changes. Here, miR-429 expression was evaluated at both acute and chronic exposure to salt. To accomplish this, fish were acclimated to FW and acutely exposed for 24 h to seawater (SW, 30 g.L^−1^) to evaluate the responses of expression of miR-429 by RT-qPCR in the gills, brain, liver, and kidney. Then, after verifying where the differential expression was present in a challenge situation, the fish were submitted to a protocol of milder salinity alterations and with evaluation of miR-429 expression for 15 days. Thus, fish were acclimated to FW and brackish water (BW, 10 g.L^−1^). They were then transferred to the opposite salinity treatment. We mainly discuss the role of miR-429 in stress tolerance and osmoregulation responses triggered by the osmotic changes.

## Materials and methods

### Animals and conditions

The silversides *O. humensis* used in this study came from eggs collected in nature, from a freshwater lagoon called Mirim Lagoon (Arroio Grande, Brazil 32°09′21.8″S 53°01′27.6″W). The eggs were transported to the laboratory and hatched in tanks. Fish were 1.5 years old and had a mean weight of 23.0 ± 19.3 g at the end of Experiment 1 and 27.3 ± 9.5 g at the end of Experiment 2. Therefore, the silversides were kept within an experimental room, maintained in 1,000 L cylindrical plastic tanks, and fed three times a day with commercial feed (Supra, 38% crude protein). The tank sides were opaque to reduce visual stress and the incidence of the natural luminosity was by the top. A natural photoperiod of 11 h light/13 h dark was applied. The dissolution of non-iodized sea salt in the water was performed to achieve the desired salinity levels. The acclimation period was 4 weeks for silversides of both experiments (described below). The water quality parameters of the experimental times did not differ statistically from those observed in the acclimation period, except for the salinity of the group exposed to different salinities.

The water of Experiment 1 was maintained with pH 7.00 ± 0.6; temperature 13.4 ± 1.8°C; dissolved oxygen 9.7 ± 0.6 mg.L^−1^; total ammonia levels lower than 0.6 ± 0.2 mg.L^−1^; and salinity 3.2 ± 0.7 g.L^−1^ (exclusively on acclimation period). The water of Experiment 2 was maintained with pH 7.78 ± 0.03; temperature 20.01 ± 0.34°C; dissolved oxygen 7.8 ± 0.3 mg.L^−1^; and total ammonia levels lower than 0.6 ± 0.2 mg.L^−1^. At the experimental time, the animals continued to be fed three times a day and once a week the water of the tanks was renewed. The salinity levels were achieved through the dissolution of non-iodized salt in the water.

### Experimental designs

#### Experiment 1: osmotic challenge and search of tissues where miR-429 is differentially expressed

First, a tissue screening was performed to identify where miR-429 would be modulated by the osmotic challenge. For this purpose, the silversides were separated in two groups and distributed in six tanks with three fish each (two groups; three tanks/group; three fish/tank). Both of the groups were acclimated according to what was already described. In the treated group, the salinity was then increased to 30 g.L^−1^ by adding hypersaturated water containing non-iodized sea salt in dissolution.

The control group had only water added to the tanks in the same amount as the salt-treated tanks. The fish were maintained in these conditions for 24 h until the sample collection. In total, 18 silverside fish were analyzed in Experiment 1.

#### Experiment 2: monitoring of miR-429 expression in the gills over time

After verifying that the gills were the tissues that showed differential expression of miR-429, other fish were submitted to Experiment 2. The experiment was performed in quadruplicate, totaling two groups and eight tanks with 12 fish each (two groups; four tanks/group; 12 fish/tank). Four tanks contained silversides acclimated to FW and four tanks contained silversides acclimated to BW. Subsequently, both FW and BW groups were transferred to opposite salinity treatment. Fish samples were collected at four different time points by collecting three fish per tank: a control before the osmotic transfer (D0) and on day 1 (D1), on day 7 (D7), and on day 15 (D15) after the transfer. In total, 96 silverside fish were analyzed at distinct times after salt treatment in Experiment 2.

### Sample collection and RNA extraction

When captured, at all-time points, silversides were anesthetized by immersion in water with benzocaine at 50 mg.L^−1^. While anesthetized, the fish were weighed and euthanized by medullary section and excision of the brain. In Experiment 1, the brain, gills, kidney, and liver from silversides of both groups were collected. In Experiment 2, only the gills of the silversides were collected. All of the experimental procedures were approved by the Ethics Committee on Animal Experimentation of the Federal University of Pelotas (Process no. 23110.007018/2015-85).

The tissues were preserved in liquid nitrogen (N2), and posterior stored at −80°C upon further RNA preparation. Total RNA isolation was performed using the RNeasy™ Mini Kit (Qiagen, United States), as described per the manufacturer’s description. RNA samples were treated with DNA-Free™ Kit (Invitrogen™, United States) to remove DNA contamination. Subsequently, the RNA concentration and quality were measured by UV-light spectrophotometry using NanoVue™ Plus equipment (GE Healthcare Life Sciences, United States), and only samples displaying high purity (OD_260/280_ ≥ 2.0 nm) were used in reverse transcription (RT) reactions. Additionally, RNA quality was measured on the 4200 TapeStation (Agilent Technologies, United States) through the TapeStation analysis application.

### MicroRNA cDNA synthesis

The generation of cDNA was carried out by using the TaqMan MicroRNA reverse transcription kit (Applied Biosystems; Thermo Fisher Scientific, Inc., United States), following the manufacturer’s protocol for microRNA reverse transcription. Each microRNA cDNA synthesis reaction contained 10 ng of extracted total RNA, 50 nM stem-looped RT primer, 1 × RT buffer, 0.25 mM each of dNTPs, 3.33 U/µl Multiscribe reverse transcriptase, and 0.25 U/µl RNase Inhibitor. The thermocycling conditions were as follows: 30 min at 16°C, 30 min at 42°C, and 5 min at 85°C. Thereafter, all samples were stored at −20°C until further use.

### Expression of miR-429 analysis by two-step RT-qPCR

For the two-step RT-qPCR assay, amplification was carried out using sequence specific primers for miR-429 on the Agilent Mx3005P system (Agilent Technologies, United States). The 20 µl reaction included 1.33 µl RT product, 1 × TaqMan^®^ Universal PCR Master Mix (catalog number 4324018, Applied Biosystems, United States), and 1 × TaqMan^®^ MicroRNA assays (catalog number 4427975, Applied Biosystems, United States). The reactions were incubated in a 96-well optical plate at 95°C for 10 min, following by 40 cycles of 95°C for 15 s and 60°C for 1 min. Histone h3a mRNA (*h3a,* GenBank accession number KX060037) (Silveira et al., 2018a) was used as a reference to obtain the relative fold-change in miR-429 expression in target samples using the 2^-∆∆Ct^ method.

### Statistical analysis

The Shapiro-Wilk test was used to test the normality of the data. The homogeneity of variances was evaluated by Levene’s test, O’Brien’s test, and the Brown and Forsy test. We analyzed the effect of tanks on miR-429 expression results using a factorial analysis of variance test and did not find a significant effect (*p* > 0.05) of tanks in relative expression results. When the data did not show normal distribution or homogeneous variances, data transformation was performed using the Log or Box-Cox methods, with the aim of meeting the requirements for the use of parametric tests, but without success. Therefore, the miR-429 expression in the different tissues from fish used in Experiment 1 was evaluated by Mann-Whitney test. The miR-429 expression in gills from fish used in Experiment 2 were analyzed by two-way analysis of variance (ANOVA), followed by Tukey’s posttest. All the applied statistical analyses in the present study used a confidence level of 95%.

## Results

### Expression of miR-429 after acute exposition to seawater

**FIGURE 1:**
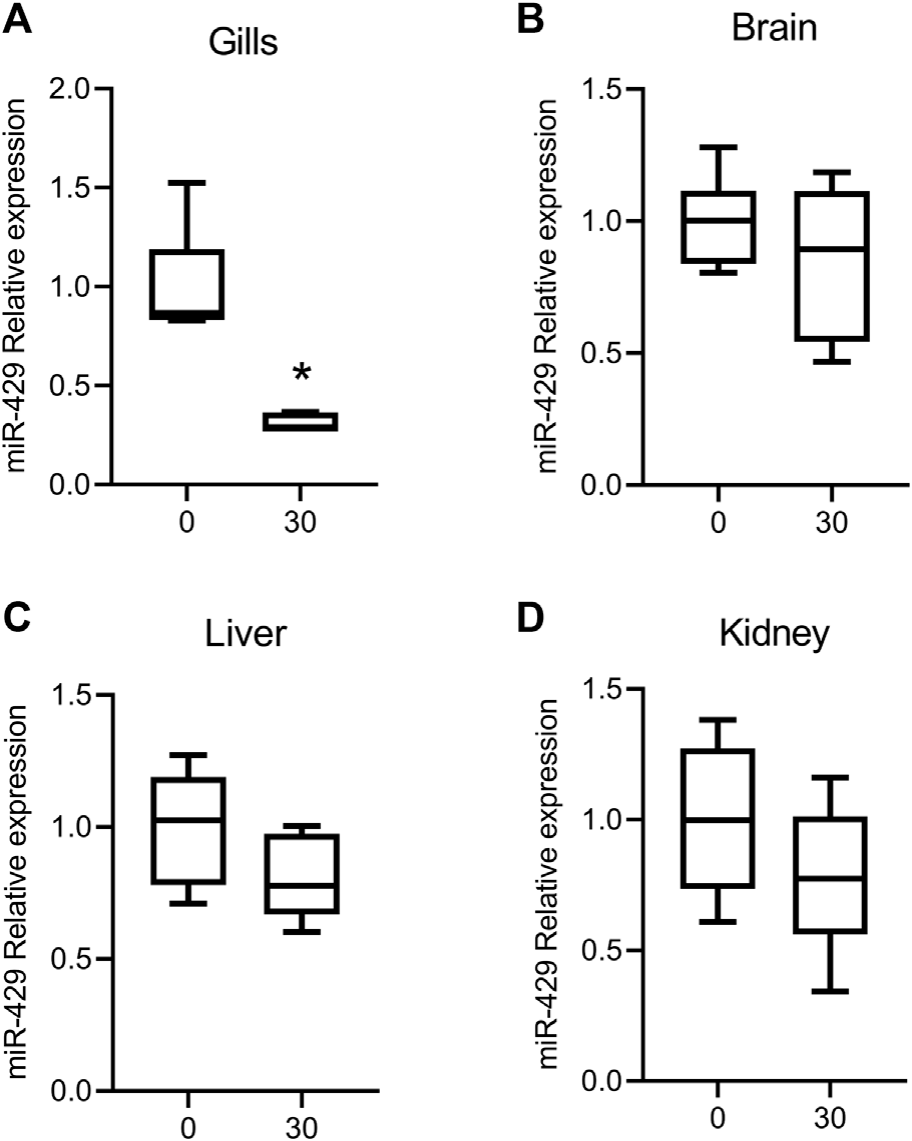
Relative expression of miR-429 in gills **(A)**, brain **(B)**, liver **(C)**, and kidney **(D)** from *Odontesthes humensis* in response to osmotic stress. The downregulation of miR-429 in gills was detected 24 h after 30 g.L^−1^ of salt using two-step RT-qPCR. The group exposed to 0 g.L^−1^ was taken as the control group. The box extends from the 25th to 75th percentiles, while the whiskers go down to the smallest value and up to the largest. The data was expressed as the relative change compared with the control group. Asterisk (∗) indicates significant difference compared with the control group (∗*p* < 0.05).

The relative expression of miR-429 in the brain, liver, and kidney did not differ between SW- and FW-exposed fishes (*p* > 0.05) (Figures 1B–D). However, a notorious downregulation of miR-429 in gills was observed (*p* < 0.05) after 24 h of exposition to 30 g.L^−1^ of salt (Figure 1A). The observation that these tissues were the locality where the miR-429 was differentially modulated after acute osmotic challenge allowed the selection of the gills as a target for long-term evaluation.

### Expression of miR-429 after mild salinity changes

**FIGURE 2:**
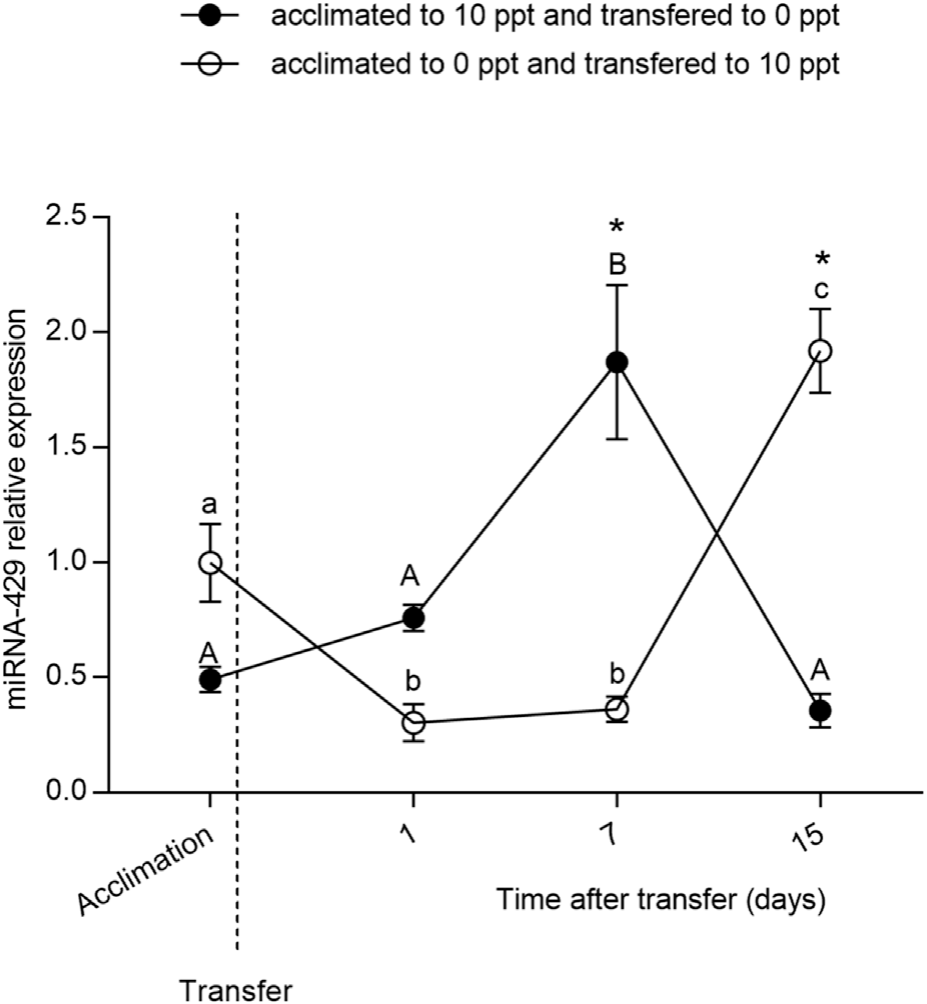
Relative expression of miR-429 from *Odontesthes humensis* submitted to salinity transfer. Different letters in each group (lowercase for freshwater (FW) to brackish water (BW) transferred group and uppercase for BW–FW transferred group) represent significant differences between the measurements in the same group. The asterisk (∗) represents the significant difference between groups in a same experimental time (∗*p* < 0.05). The vertical broken line indicates the transfer from BW to FW and from FW to BW moments.

The relative expression of miR-429 (Figure 2) did not differ between FW- and BW-acclimated fish at D0 (*p* > 0.05). Nevertheless, a change in miR-429 expression was observed after the FW-BW transfer. In both FW-BW- and BW-FW-transferred groups, miR-429 expression remained without a difference at D1. The miRNA expression significantly increased (*p* < 0.05) at D7 in the BW-FW transfer and remained without a difference at D15 (*p* > 0.05). The miR-429 expression level did not change in the FW-BW-transferred fish (*p* > 0.05) at D1 until D7. However, there was a notable increase (*p* < 0.05) of miR-429 at D15.

## Discussion

The silverside *O. humensis* is a teleost that moves from coastal waters near land or in estuaries to near-shore shallow areas. Due to their recent marine origin, and because their currently inhabit both freshwater and brackish waters environments, silversides can tolerate a wide range of environmental salinity and are regarded as euryhaline (Bemvenuti, 2006). The silversides have received attention from researchers who aim to develop the use of fish farming in estuarine regions, where there is a constant variation in the salinity of water available for use in cultivation, and in regions of continental farming, where the available water is brackish (Piedras, 2009; Heras And Roldán, 2011).

As a wild species, *O. humensis* reside directly in water environments and usually experience sudden changes or frequent fluctuation of environmental salinity. Indeed, salinity is a frequent abiotic stressor that restrains fish growth and development by triggering osmotic stress responses. Thus, when fishes are subjected to salinity stress, related genes are activated to induce tolerance to the salinity-mediated stress (Silveira et al., 2018b). Although miRNAs provide fish with elaborate strategies for gene regulation at the post-transcriptional level, the precise mechanisms by which miRNAs employ their regulatory function facing osmotic stress are not fully understood. Therefore, the role of miRNAs in osmoregulation has attracted much attention from researchers.

Similarly to fishes, soil salinity is a major abiotic stress in plant agriculture, where high levels of salt can induce ion imbalance and hyperosmotic stress. Indeed, salinity alters miRNA expression in tobacco in a dose-dependent manner because miR-395 is found to be severely sensitive to salt (Frazier et al., 2011). The expression of the miR-169 family members is thought to be inducible by salinity because they regulate several genes involved with salt tolerance in *Arabidopsis thaliana* and transgenic rice (Zhao et al., 2009). Moreover, oni-miR-30c acts limit the expression of *hsp70* gene, which is regarded as essential in osmoregulation in tilapia (Yan et al., 2012b). In addition, the miR-8 family, expressed mainly in ionocytes, is directly attached to osmoregulation in zebrafish*.* Furthermore, the miR-8 family acts by regulating the expression of Nherf1, which regulates transmembrane ionic transport (Flynt et al., 2009).

Interestingly, regarding the gills as a crucial regulator of internal osmotic balance, miR-429 seems to be more copious in gills than in any other organ. This indicates that the gills play a pivotal role in regulating environmental salinity tolerance in a post-transcriptional level *via* miRNAs (Bartel, 2009). Furthermore, miR-429 is known to compose a regulatory circuit that allows fish to respond to osmotic stress because it directly regulates the expression of osmotic stress transcription factor 1 (Ostf1) by targeting its 3′-UTR region (Fiol et al., 2006; Yan et al., 2012b).

Given that there are no studies that relate the epigenetic regulation of osmotic stress through miRNAs in silversides, we have pioneered the analysis of miRNAs relative expression under environmental salinity using *O. humensis* as a bioindicator. Here, the expression of miR-429 analysis by Two-step RT-qPCR confirms our hypothesis that this miRNA is directly involved in the osmoregulation acclimation process in silversides and emerges as osmotic stress marker because miR-429 presented significant differential gene expression under environmental salinity.

In this study, we first performed an acute exposure to SW (30 g.L^−1^) for 24 h to determine in which tissue miR-429 is differentially expressed during hyperosmotic stress. The miR-429 expression pattern was found to be remarkably decreased in the gills after the acute exposure period. These results reveal that miR-429 has a prime role in silversides osmoregulation following acute salt treatment. Similar effects in miR-429 expression were observed in Nile tilapia challenged by salinity stress, which presented lower expression levels of miR-429 in gills after hyperosmotic situations (Yan et al., 2012a).

We subsequently exposed the fish previously acclimated to FW to a salt concentration of 10 g.L^−1^ for 15 days (and *vice versa*) to evaluate the role of miR-429 in osmoregulation. A milder concentration of salt was used because the animals would not be able to stand the SW for such a long period (unpublished personal observations). In BW-FW-transferred fish, miR-429 expression increased at D7 upon salinity stress. Likewise, in FW-BW-transferred fish, miR-429 significantly increased at D15. Taken together, these findings indicate that miR-429 in silversides plays a key regulatory role in regulating salinity tolerance at both acute and chronic exposition.

In addition to being regarded as FW, silversides are often exposed to salt and brackish water on estuaries and coastal lagoons of South America, and have better development and survival in saline environments (Tsuzuki et al., 2001). Previous literature has observed that the BW-FW transfer is more stressful to *O. humensis* than the FW-BW transfer (Silveira et al., 2018b). In this sense, given that in BW-FW group the expression of miR-429 increased after 1 week of acclimation, more precociously than in FW-BW group, miR-429 directly regulates silversides osmoregulation. In FW-BW-transferred fish, miR-429 expression level significantly increased at D15. This indicates that this miRNA also plays a key role in long-term response to salinity.

The levels of salinity were shown to modulate the energy supply available for growth and reproduction in farmed fishes (Chand et al., 2015), and its optimal adjustment can benefit *O. humensis* production in captivity for aquaculture purposes. Indeed, the brackish medium is ideal for the cultivation of silversides. Cultivating this species in brackish environments, which are near-isosmotic environments, has vast potential in aquaculture to reduce economic losses by mortality due to the handling and transport of the fish, and due to the low quality of the water (Tsuzuki et al., 2001). In the same way, the culture of the silversides in brackish medium makes it possible to increase the survival rate of the embryos, increase the growth rates and feed conversion levels, and improve the efficiency of energy absorption and other parameters of productive interest (Silveira et al., 2018b).

In summary, our results suggests that miR-429 in silversides plays a prime role in osmoregulation and is mainly expressed in the gills during osmotic stress. Our findings suggest that miRNAs appear to regulate salt tolerance at the post-transcriptional level. Therefore, miR-429 has substantial potential to be developed as a novel molecular marker to assist silverside aquaculture by farming this fish in estuarine regions and brackish environments. It can also be used in the selection based on epigenetic markers of salinity-tolerant fish. To understand the integrated responses and regulatory roles of miR-429 during osmotic stress in silversides, further studies that aim to analyze the gene expression of target genes modulated by miR-429 are required. Finally, our study provides evidence for the use of *O. humensis* as a model in scientific research.

## Data Availability

The original contributions presented in the study are included in the article/supplementary material, further inquiries can be directed to the corresponding author.
